# Chemotherapy-related adverse effects with anthracycline and taxane-containing regimens in patients with localized Breast cancer: a descriptive study: Mohammed VI University Hospital, Medical Oncology Department, Marrakech, Morocco

**DOI:** 10.1186/s12885-023-11616-5

**Published:** 2024-01-02

**Authors:** Farah Choulli, Hassan Abdelilah Tafenzi, Faiçal EL Hattimy, Mohamed Khaled Choulli, Rhizlane Belbaraka

**Affiliations:** 1 Medical Oncology Department, Mohammed VI University Hospital of Marrakech, Marrakech, Morocco; 2 Academic Health Observatory, Biosciences and Health Laboratory, Faculty of Medicine and Pharmacy, Cadi Ayyad University, Marrakech, Morocco; 3 Genetics and Biometry Department, Faculty of Sciences, Ibn Tofail University, Kenitra, Morocco

**Keywords:** Localized Breast cancer, Chemotherapy-related adverse effects, Anthracycline and Taxane-containg regimens, Listening approach, Structured interview, Morocco

## Abstract

**Background:**

Although the side effects of chemotherapy are frequently described in research studies, there is little evidence on how common they are in everyday clinical care. This study’s goal was to assess the most prevalent short-term side effects experienced by patients with localized breast cancer, undergoing chemotherapy based on anthracyclines and taxane-containing treatments, at the medical oncology department of the Mohammed VI University Hospital of Marrakech, Morocco.

**Methods:**

This was a descriptive study. We conducted a listening session at the outpatient department of the hospital with the help of a structured questionnaire. The session engaged 122 women who had undergone cycles of chemotherapy. A chi-square test was used to compare the incidence and relative risk of short side effects with both anthracycline and taxane-containing regimens.

**Results:**

The average age of participants was 49.1 years. In both regimens, the findings highlighted the frequency and relative risk of the following adverse effects: systemic symptoms (fever, asthenia and sleep disorder), gastrointestinal toxicity (Vomiting, nausea, diarrhoea, constipation, mucositis and loss of appetite), dermatological toxicity (Skin reactions on hands/feet, nail toxicity, allergies, alopecia and peripheral edema), neurological toxicity (neuropathy), arthromyalgia and ocular toxicity.

**Conclusions:**

In conclusion, it is crucial for healthcare professionals to be conscious of the significance of these adverse effects. They must also know how to manage them. Likewise, the listening approach highlights its importance in the daily follow-up and monitoring of patients.

## Background

Breast cancer (BC) is the most common cancer in women worldwide [1]. Aside population aging, rising BC rates are associated with increasing social and economic development and a more Westernized lifestyle (e.g., increased smoking, poor diet, sedentary lifestyle, and reproductive changes) [2]. According to the World Health Organization (WHO), in 2020, BC affected 2.3 million people all over the world, with 6.8 million deaths [3]. Most deaths occur in low- and middle-income countries, where most patients with BC are diagnosed at very late stages due to lack of information on early detection and inadequate access to health care [4].

Every year, approximately 3.5 million Moroccans are diagnosed with BC, and this figure is expected to rise dramatically over the next decade [5]. According to the United Nations Human Development Index, Morocco has a Human Development Index (HDI) of 0.686, putting it in the medium category [6]. Its population is expected to reach 36.92 million in 2022, with a life expectancy of 77.21 years [7, 8]. Recent data are scarce, but in 2012, Morocco recorded about 2878 BC deaths [9]. And according to a report published by the Grand Casablanca Cancer Registry, BC was the most common cancer among Moroccan women in 2016, accounting for 35.8% of all new cancers, with age-standardized rates of 49.5/100 000 [5, 9].

However, despite the upsurge of BC cases, Morocco has made significant efforts to track the global progress of medical advancements. Several treatments, including conventional chemotherapy, hormone therapy, targeted drug therapy, and immunotherapy, have been introduced over time and have considerably increased survival [10]. Nevertheless, since all drugs have side effects, monitoring patients after each cycle of their treatment remains essential.

In this context, the follow-up of patients with localized BC treated with neoadjuvant or adjuvant chemotherapy with anthracycline and taxane-containing regimens caught our attention. These treatments should be carefully considered since they may have various cytotoxic or adverse effects immediately, as well as in the short- and long terms (e.g., fatigue, alopecia, gastrointestinal disorders, mucositis, etc.) [11]. Additionally, many side effects may be avoided or reduced if thorough assessments were carried out following each cycle of treatment [12].

In our medical oncology department at the Mohammed VI University Hospital of Marrakech, and despite all efforts made by the medical staff, unfortunately, it is still challenging to provide constant and accurate follow-up of patients after each chemotherapy cycle. Thus, to overcome this problem, we initially introduced a new listening session, which consists of having direct interviews with patients in order to perceive and record their personal experience of chemotherapy and its side effects. To this end, a social officer coordinates perfectly with the medical staff to manage the daily schedule for monitoring and listening to the patients.

This social and direct listening approach proved quite significant in the continuous follow-up of patients. Therefore, we are thinking of developing an artificial intelligence (AI) mobile app for remote monitoring and follow-up of Moroccan BC patients experiencing adverse effects of chemotherapy.

The findings in this article were gathered from medical records and daily assessment of the short-term side effects experienced by the patients themselves.

## Methods

### Study design and setting

A structured interview method was adopted in this study to understand the conditions of Moroccan women patients, determine patients’ personal experiences and side effects of chemotherapy, and cultivate a deeper awareness of a specific problem. Anthracyclines and taxane-containing drugs were used to study patients with localized breast cancer receiving neoadjuvant or adjuvant chemotherapy. The study took place in the medical oncology department of the Mohammed VI University Hospital in Marrakech, Morocco, from September 2021 to March 2022.

### Participants

With the help of selected inclusion criteria, 122 women with localized breast cancer receiving neoadjuvant or adjuvant chemotherapy with anthracyclines and taxane-containing regimens, and having completed at least one cycle under this protocol, were included in the study. All patients who met the inclusion criteria had already responded to the survey created for the study. Patients with metastatic BC, and those starting their first chemotherapy cycle were excluded.

### Study tool

In order to research and probe questions related to the study objectives, a structured interview topic guide was used as a framework. This guide covered all of the common non-hematological short-term side effects associated with the antracycline and taxane-containing regimens. Doxorubicin, epirubicin, cyclophosphamide, docetaxel and weekly paclitaxel were all included in different combinations with the anthracyclines and taxanes. The interview focused on participants’ perspectives on chemotherapy, including their personal experiences with the disease, its adverse effects, and their use of complementary and alternative therapies. The researchers discussed the first draft of the guide about interview topics and conducted pilot interviews to ensure that it was clear and efficient in locating the necessary data.

### Procedure

Patients had to pre-register for an oncology consultation prior to receiving treatment. They were then admitted at the outpatient department for a predetermined amount of hours, during which time staff members provide specialized medical care. These procedures inside the hospital involve equipment handling and supervision. At the outpatient department, each patient was questioned about any toxicities they may have encountered throughout the previous cycle or the preceding 21 days. The questionnaire was randomly distributed during one of the six or eight treatment cycles. Patients were able to freely express themselves in their own way. At the end, they were omitted from subsequent cycles. Each interview typically lasted between 15 and 30 min. Before the trial, the participants provided oral informed consent after receiving all the required information and having any questions answered.

### Data analysis

Chi-square test was used to compare the existence of difference and risk between each side effect of both regimens. A p-value less than 0.05 is considered to be statistically significant. All statistical analysis were done using IBM SPSS Statistics software version 26,0.

## Results

### Participant profile

The final sample included 122 women with localized BC, who had undergone cycles of chemotherapy. The study’s participants were women with an average age of 49.1 years (Ranging from 28 to 80), who were married (67.2%), with children (73.77%), and beneficiaries of the Medical Assistance Scheme (91%). In terms of education, 71.3% were illiterate and were usually accompanied during the consultation.

### Clinical and pathologic characteristics

Each patient’s medical record served as the basis for our analysis, which allowed us to determine the clinical and pathologic characteristics, taking into consideration the possibility that the records sometimes contain missing or incomplete data (Table 1). In this study, 86.9% of patients had Invasive Ductal Carcinoma (IDC) type BC. Among the 122 cases, 20.5% had Triple Negative Breast Cancer (TNBC), 59.02% were HER2-negative, and 60.7% were Hormone Receptor (HR) positive. Localization in the left breast was the most common and represented 56.6% of cases. The majority of patients had grade II BC (52.5%), absence of lymph nodes N0 (32.8%) and T_2_ size tumors (43.4%). With a score 1 of Performance Status (PS), 92.6% of patients could perform light tasks. The proportion of patients receiving adjuvant therapy was 63.9%, while 32.8% received neoadjuvant therapy.

**Table 1:** Clinical and pathologic characteristics in the study population

		Number % of patients
Histological type	IDC	(106) 86.9%
	ILC	(12) 9.8%
	Missing values	(4) 3.3%
Breast	Left	(69) 56.6%
	Right	(46) 37.7%
	Missing values	(7) 5.7%
Hormone receptor status (HR)	Positive	(74) 60.7%
	Negative	(42) 34.4%
	Missing values	(6) 4.9%
HER2 status	Positive	(44) 36.1%
	Negative	(72) 59.02%
	Missing values	(6) 4.9%
HR-/HER2-	Triple negative	(25) 20.5%
Histological grade	SBR I	(4) 3.3%
	SBR II	(64) 52.5%
	SBR III	(29) 23.8%
	Missing values	(25) 20.5%
Tumor size	T_1_	(20) 16.4%
	T_2_	(53) 43.4%
	T_3_	(18) 14.8%
	T_4_	(24) 19.7%
	Missing values	(7) 5.7%
Lymph Nodes	N_0_	(40) 32.8%
	N_1_	(37) 30.3%
	N_2_	(28) 23.0%
	N_3_	(10) 8.2%
	Missing values	(7) 5.7%
Performance Status (PS)	1	(113) 92.6%
	2	(7) 5.7%
	3	(2) 1.6%
Adjuvancy	Neoadjuvant	(40) 32.8%
	Adjuvant	(78) 63.9%
	Missing values	(4) 3.3%

Abbreviations : IDC = Invasive ductal carcinoma ; ILC = Invasive lobular carcinoma ; HER2 = Human Epidermal growth factor Receptor 2 ; SBR = Scarff-Bloom-Richardson

### Adverse effects of anthracyclines and taxanes

Adverse effects that were considered in the study included systemic, gastrointestinal, dermatological and neurological symptoms, arthromyalgia, and ocular toxicities. Table 2 lists the rates of toxicities associated with the two regimens, along with the significance of the comparisons and the relative risk.

**Table 2:** Anthracycline and taxane-related short-term side effects: frequency, significance, and relative risk (RR)

		Anthracyclines		Taxanes				
		(n) % Yes	(n) % No	(n) % Yes	(n) % No	χ2	p-value	RR (95% CI)
Systemic symptoms	Fever	(19) 15,6%	(103) 84,4%	(19) 15,6%	(103) 84,4%	,000^a^	1,000	1,000(1,000–1,998)
	Asthenia	(97) 79,5%	(25) 20,5%	(65) 53,3%	(57) 46,7%	18,809^a^	< 0,001	3,402(1,933-5,989)
	Sleep disorder	(34) 27,9%	(88) 72,1%	(30) 24,6%	(92) 75,4%	,339^a^	0,560	1,185(0,669-2,098)
Gastrointestinal toxicity	Vomiting	(107) 87,7%	(15) 12,3%	(22) 18,0%	(100) 82,0%	118,834^a^	< 0,001	32,424(15,931 − 65,994)
	Nausea	(101) 82,8%	(21) 17,2%	(42) 34,4%	(80) 65,5%	58,808^a^	< 0,001	9,161(5,026-16-699)
	Diarrhoea	(39) 32,0%	(83) 68,0%	(39) 32,0%	(83) 68,0%	,000^a^	1,000	1,000(0,584-1,713)
	Constipation	(39) 32,0%	(83) 68,0%	(17) 13,9%	(105) 86,1%	11,217^a^	< 0,001	2,902(1,533-5,494)
	Mucositis	(70) 57,4%	(52) 42,6%	(43) 35,2%	(79) 64,8%	12,016^a^	< 0,001	2,473(1,476-4,145)
	Loss of appetite	(99) 81,1%	(23) 18,9%	(33) 27,0%	(89) 73,0%	71,893^a^	< 0,001	11,609(6,343 − 21,247)
Dermatological toxicity	Skin reaction of Hands/feet	(24) 19,7%	(98) 80,3%	(35) 28,7%	(87) 71,3%	2,705^a^	0,100	0,609(0,336-1,103)
	Nail toxicity	(74) 60,7%	(48) 39,3%	(47) 38,5%	(75) 61,5%	11,952^a^	0,001	2,460(1,470-4,116)
	Allergies	(19) 15,6%	(103) 84,4%	(53) 43,4%	(69) 56,6%	22,776^a^	< 0,001	0,240(0,131-0,440)
	Alopecia	(118) 96,7%	(4) 3,3%	(116) 95,1%	(6) 4,9%	,417^a^	0,518	1,526(0,420-5,548)
	Peripheral edema	(5) 4,1%	(117) 95,9%	(19) 15,6%	(103) 84,4%	9,058^a^	0,003	0,232(0,084 − 0,643)
Neurological toxicity	Peripheral neuropathy	(15) 12,3%	(107) 87,7%	(91) 74,6%	(31) 25,4%	96,346^a^	< 0,001	0,048(0,024 − 0,094)
Arthromyalgia	Arthromyalgia	(8) 6,6%	(114) 93,4%	(76) 62,3%	(46) 37,7%	83,948^a^	< 0,001	0,042(0,019 − 0,095)
Ocular toxicity	Eye toxicity	(18) 14,8%	(104) 85,2%	(44) 36,1%	(78) 63,9%	14,618^a^	< 0,001	0,307(0,165-0,572)

#### Anthracyclines

With a joint rate of 79.5% for the three systemic symptoms (fever, asthenia, and sleep disorder), asthenia was the most prevalent in patients. Most of the patients who experienced gastrointestinal toxicity reported episodes of vomiting (87.7%), nausea (82.8%), constipation (32.0%), and appetite loss (81.1%). Of those, 57.4% had mucositis. Alopecia, or hair loss, is one of the most commonly reported dermatological symptoms, with the highest index of 96.7%. While 60.7% of patients complained about nail color changes. There were only a few cases of neurological toxicity (12.3%), arthromyalgia (6.6%), and ocular toxicity (14.8%).

#### Taxanes

Asthenia continued to be the most common symptom among the systemic symptoms with an index of 53.3%. Gastrointestinal toxicities were less frequently reported, vomiting (18%), nausea (34.4%), constipation (13.9%), and loss of appetite (27.0%). Alopecia and allergies were prevalent in 95.1% and 43.4% of patients, respectively. Peripheral neuropathy affected 74.6% of individuals, while arthromyalgia affected 62.3%. Last but not least, 36.1% reported ocular toxicity.

#### Relative risk and comparisons of the incidence of toxicities associated with the two regimens

In order to highlight their influence, we estimated the relative risk (RR) of each toxicity associated with anthracyclines and taxanes. When compared, the most substantial higher risks were noted for vomiting, nausea and appetite loss. The RR for vomiting was estimated at 32.424 (95% CI 15.931–65.994; p < 0.001; χ2 = 118.834^a^), nausea at 9.161 (95% CI 5.026–16.699; p < 0,001; χ2 = 58.808^a^), and the RR of appetite loss at 11.609 (95% CI 6.343–21.247; p < 0.001; χ2 = 71.893^a^). Anthracyclines were also associated with a significantly increased RR of asthenia (RR 3.402, 95% CI 1.933–5.989; p < 0.001; χ2 = 18.809^a^), constipation (RR 2.902, 95% CI 1.533–5.494; p < 0.001; χ2 = 11.217^a^), mucositis (RR 2.473, 95% CI 1.476–4.145; p < 0.001; χ2 = 12.016^a^), and nail toxicity (RR 2.460, 95% 1.470–4.116, p < 0.001; χ2 = 11.952^a^). However, the incidence of allergies (RR 0.240, 95% CI 0.131–0.440; p < 0.001; χ2 = 22.776^a^), peripheral edema (RR 0.232, 95% CI 0.084–0.643; p = 0.003; χ2 = 9.058^a^), neuropathy (RR 0.048, 95% CI 0.024–0.094; p < 0.001; χ2 = 96,346^a^), arthromyalgia (RR 0.042, 95% CI 0.019–0.095; p < 0,001; χ2 = 83,948^a^), and ocular toxicity (RR 0.307, 95% CI 0.165–0.572); p < 0,001; χ2 = 14,618^a^), were lower in anthracyclines compared to taxanes. The other toxicities (fever, sleep disorder, diarrhoea, skin reaction of hands/feet, and alopecia) did not have a higher impact expressed by RR.

#### Variation in side effects between the two regimens

**Fig. 1:**
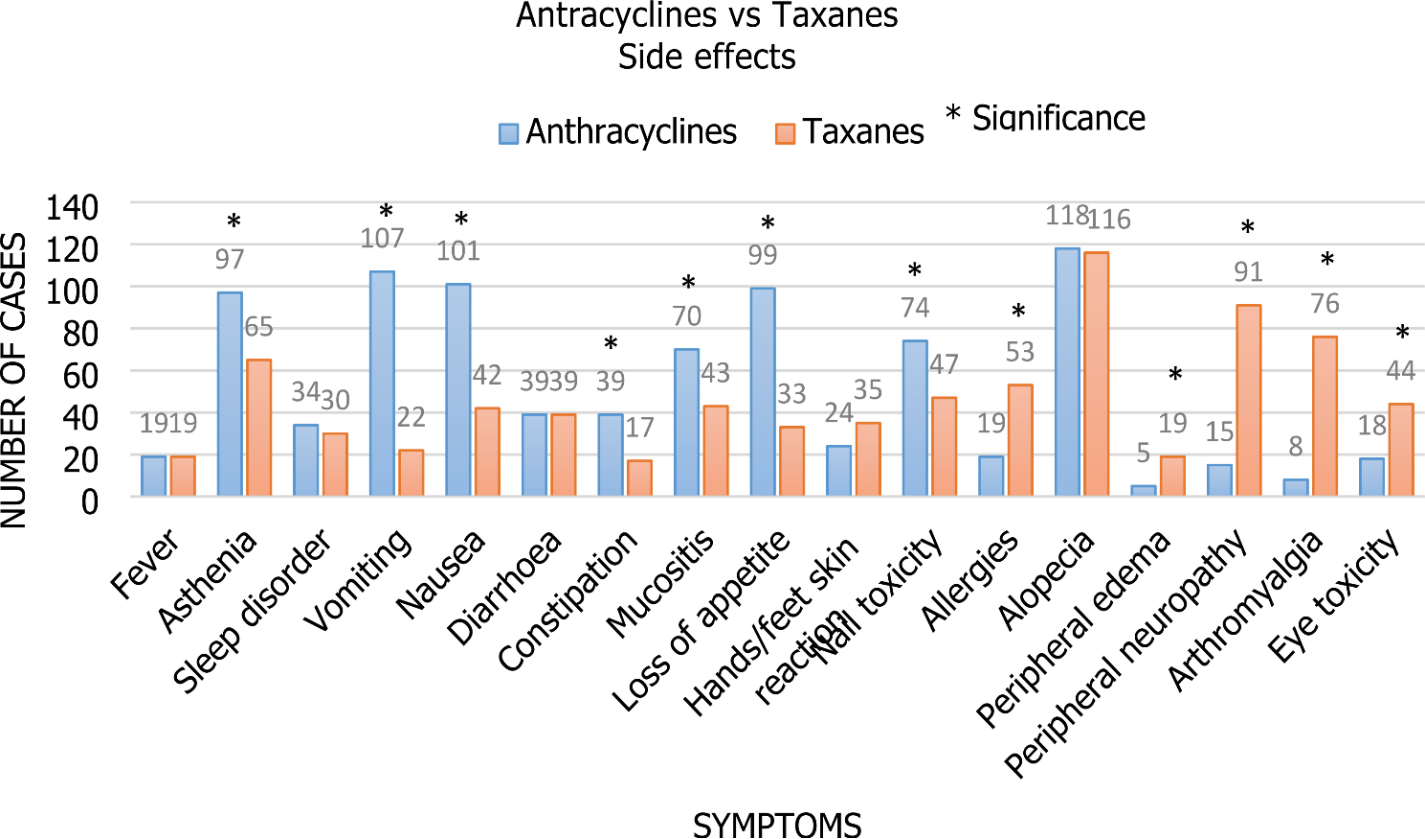
The variations in the short-term side effects between the two regimens. When taking Anthracyclines, patients reported having more asthenia (n = 97), vomiting (n = 107), nausea (n = 101), constipation (n = 39), Mucositis (n = 70), appetite loss (n = 99), and nail color changes (n = 74). Increased allergies (n = 53), edema (n = 19), neuropathy (n = 91), Arthromyalgia (n = 76), and ocular toxicity (n = 44) were observed with the use of Taxanes

The differences in these short-term side effects between the two regimens are shown in Fig. 1. Patients have increased asthenia, vomiting, nausea, constipation, mucositis, appetite loss, and changes in nail color when taking anthracyclines. With the usage of taxanes, increased allergies, edema, neuropathy, arthromyalgia, and ocular toxicity were noted.

## Discussion

As BC remains a global health problem, it is indeed an urgent priority. This is why in-depth studies concerning it must be regularly considered. Thus, knowing different aspects of the disease is essential.

With an emphasis on presenting details on the actual conditions at the oncology unit of the Mohammed VI University Hospital in Marrakech, Morocco, our aim was to give a descriptive overview of BC in our nation. In order to achieve this, we provided data on participants’ profiles as well as clinical and pathologic characteristics. Then, by employing a new listening approach, we assessed the adverse effects reported by patients with localized BC undergoing chemotherapy with anthracyclines and taxanes.

Our findings suggest that one of the key elements that must be included in the patient care process is the deployment of this new strategic approach of directly listening to patients at the outpatient clinic. We were able to clearly understand from the interviews, which lasted 15 to 30 min on average, how important it is to set up daily follow-up sessions with these patients since, deep down, they all recognized that they needed it. Patients were allowed to freely express themselves, and by telling us about how they deal with their illness on a daily basis, they were able to feel more at ease and confident. Additionally, after receiving initial cancer therapy, the importance of this daily patient monitoring session would also depend on a number of other factors, including the early detection of relapses, ongoing treatment monitoring, the management of side effects, and the identification of long-term treatment effects [13].

Therefore, despite the challenging circumstances including the growing number of BC patients receiving care, the lack of time, and occasionally even the stock-outs of therapies in our oncology unit, medical staff continue to do everything in their power to meet the needs of their patients. Consequently, it makes perfect sense that under these conditions, patient follow-up on a daily basis represents a significant difficulty for our healthcare professionals. To this end, and after discussions with the team, it was decided that the best way to proceed, would be to develop a mHealth app using AI techniques for remote monitoring and follow-up of these BC patients receiving chemotherapy, in order to improve information sharing and patient-doctor communication.

The patient-listening approach was shown in many studies to be effective in promoting recovery, improving clinical outcomes, and strengthening the relationship between the patient and the clinician. Additionally, the value of follow-up in ensuring that patients receive continued supportive care by reassuring, advising, and identifying any psychosocial or practical issues [13, 14].

On the other hand, this study allowed us to set up qualitative indicators. Direct interviews were conducted with 122 women at the outpatient clinic, who had an average age of 49,1 years. This is consistent with a study conducted by El Fouhi et al. in Morocco in 2018, which concluded that individuals with BC had ages ranging from 40 to 50 years old [15]. Moreover, our observations have shown that the majority of patients were illiterate (71,3%) and benefited from the Medical Assistance Scheme (RAMED) (91%), a health program that provides assistance to poor people in rural areas, suffering from serious illness, even if the access to innovative treatments is limited for this population. These patients may also need to resort to other organizations for assistance as oncology costs are rising faster than RAMED’s ability to pay for treatment [16].

Furthermore, according to literature, 56.32% of breast tumors are frequently discovered on the left breast. This is supported by our findings, which showed that the left breast accounts for 56.6% of cases and the right one for 37.7%. With rates of 86.9% for IDC and 52.5% for SBR II, our study once again confirms their dominance in terms of the histological type and the SBR histoprognostic grade. According to Bakkach et al. the proportion of HER2-positive tumors ranged from 15.2 to 48%, whereas the rate of hormone-negative tumors was around 34% [17]. In line with this study, we found that 34,4% of cases had RH negative, and 36,1% had HER2 positive. TNBC, which is the most aggressive BC subtype, was found in 20,5% of our cases, consistent with S. Krishnamurthy et al [18].

To our knowledge, this is the first study that described the chemotherapy adverse effects that Moroccan patients with localized BC experienced. Be it at early or advanced phases of the disease, chemotherapy is still a critical component of the care given to patients with BC. Nowadays, there are numerous chemotherapy regimens available for patients with BC; however, each regimen varies in terms of its constituent agents, method of administration, frequency, effectiveness, and adverse effects [19].

Our focus was drawn to the short-term adverse effects of anthracyclines and taxanes, two chemotherapeutic agents often employed in sequential regimens in the adjuvant and neoadjuvant treatment of early BC [20]. It was demonstrated that their administration reduced BC mortality by 20–25% and the 10-year risk of BC relapse by a third [21]. Usually, in standard clinical practice, an anthracycline-based chemotherapy is given first, followed by a taxane [22].

According to our findings, when taking anthracyclines, patients were more likely to experience asthenia compared to taxanes (79.5%; p < 0.001). This finding is in line with that of a study by Peoples et al., which found a high prevalence of chemotherapy-related asthenia (58–94%), particularly at the start of treatment with regimens containing doxorubicin [23]. Vomiting (87.7%), nausea (82.8%), constipation (32.0%), and loss of appetite (81.1%) were the most distressing early gastrointestinal toxicities during the first cycles of anthracyclines (p < 0.001). Our findings are similar to those of the study conducted by Gadisa et al., which revealed that more than 88% of participants reported nausea and vomiting during treatment with anthracyclines, in comparison to taxanes. Patients also complained about nail color changes during treatment with anthracyclines in comparison to taxanes (60.7%; p < 0.001), and mucositis (57 0.4%; p < 0,001) [24].

As opposed to anthracyclines, patients were more likely to experience neurotoxicity with taxane regimens, with higher rates of neurological toxicity and arthromyalgia (74.6% and 62.3%; p < 0,001). The incidences found are in consistent with other studies [22, 23]. Furthermore, it was found that patients are also more likely to experience allergies and ocular toxicities in comparison with anthracyclines (43.4% and 36.1% ; p < 0,001).

However, in both regimens patients suffered from alopecia, with respective rates of 96,7% and 95,1%. Overall, the toxicity profile was similar to that observed by oncologists in our unit and in other trials using anthracycline-taxane regimens [25].

For further support and according to our RR results, patients on anthracyclines have been shown to have an extremely high risk of suffering from vomiting, nausea and loss of appetite. In addition, they have a threefold risk of developing asthenia, and a twice as high risk of developing mucositis, nail toxicity and constipation than taxanes.

The difference between these short-term side effects of anthracyclines and taxanes in breast cancer treatment can be explained by several factors, including their mechanisms of action: Anthracyclines interfere with DNA replication, causing DNA damage that ultimately leads to cell death; Taxanes disrupt normal microtubule function, leading to cell cycle arrest and cell death [26]. They may also be affected by patient characteristics like age, gender, underlying health (For instance, patients who already have conditions like heart disease or liver dysfunction may be more vulnerable to some side effects, particularly those related to Anthracyclines), as well as genetic factors [27]. In addition, concomitant medications, medications prescribed for the management of side effects, diet and psychosocial factors are all factors that have an impact on the overall side effect profile [28]. Therefore, it is important for healthcare professionals to assess these factors and adjust treatment plans accordingly, as well as the importance of close monitoring and direct communication between doctors and patients to manage and reduce these side effects.

Thus, it is necessary to know, collect and record the clinical performances on each of the side effects linked to each type of chemotherapy treatment [29]. Patients frequently underestimate the importance of side effects, mistaking them for a regular part of treatment or proof that treatment is “effective” [30, 31]. Additionally, they are required to report any incidents of toxicity until their next appointment, so the frequency and severity are likely to be skewed by memory [13]. This perfectly explains our decision to develop a mHealth app for the follow-up of these patients, which will also aim to reduce morbidity, unnecessary hospitalizations or the premature cessation of chemotherapy.

### Limitation of the study

It is necessary to conduct a subsequent study that includes the grading of each encountered toxicity in addition to expanding the patient population. However, hematological toxicity should also be taken into account.

## Conclusion

In conclusion, this study used a direct listening approach to emphasize some of the negative short-term side effects of chemotherapy that Moroccan women with localized BC frequently face. This listening approach proved to be helpful inside the outpatient clinic by helping women communicate and recognize their needs more efficiently. Additionally, it demonstrated the value of ongoing patient monitoring and management of these negative side effects.

### What is already known on this topic

Chemotherapy induces a wide range of adverse effects that affect all cells in the body, particularly those that share characteristics with tumor cells, such as hair follicles, cells of the digestive tract, bone marrow, and cells of the reproductive system.

### What this study adds

The situation among Moroccan women with localized breast cancer undergoing the adverse effects of chemotherapy based on anthracyclines and taxanes.

## Data Availability

The datasets used and/or analysed in the current study are available from the corresponding author upon reasonable request.

## Declarations

### Administrative support

Biosciences and Health Laboratory, Faculty of Medicine and Pharmacy, Cadi Ayyad Unicersity, Marrakech, Morocco & Medical Oncology Department, Mohammed VI University Hospital, Marrakech, Morocco.
